# A Novel Composite Material UiO-66@HNT/Pebax Mixed Matrix Membranes for Enhanced CO_2_/N_2_ Separation

**DOI:** 10.3390/membranes11090693

**Published:** 2021-09-07

**Authors:** Fei Guo, Bingzhang Li, Rui Ding, Dongsheng Li, Xiaobin Jiang, Gaohong He, Wu Xiao

**Affiliations:** 1 State Key Laboratory of Fine Chemicals, Department of Chemical Engineering, Dalian University of Technology, 2 Linggong Road, Dalian 116024, China; guofei195@mail.dlut.edu.cn (F.G.); benchwon50@163.com (B.L.); dingrui@mail.dlut.edu.cn (R.D.); lds_0531@mail.dlut.edu.cn (D.L.); xbjiang@dlut.edu.cn (X.J.); hgaohong@dlut.edu.cn (G.H.); 2 State Key Laboratory of Fine Chemicals, School of Petroleum and Chemical Engineering, Dalian University of Technology, 2 Dagong Road, Panjin 124221, China

**Keywords:** composite material, UiO-66, HNT, mixed matrix membrane, CO_2_/N_2_ separation

## Abstract

Mixing a polymer matrix and nanofiller to prepare a mixed matrix membrane (MMM) is an effective method for enhancing gas separation performance. In this work, a unique UiO-66-decorated halloysite nanotubes composite material (UiO-66@HNT) was successfully synthesized via a solvothermal method and dispersed into the Pebax-1657 matrix to prepare MMMs for CO_2_/N_2_ separation. A remarkable characteristic of this MMM was that the HNT lumen provided the highway for CO_2_ diffusion due to the unique affinity of UiO-66 for CO_2_. Simultaneously, the close connection of the UiO-66 layer on the external surface of HNTs created relatively continuous pathways for gas permeation. A suite of microscopy, diffraction, and thermal techniques was used to characterize the morphology and structure of UiO-66@HNT and the membranes. As expected, the embedding UiO-66@HNT composite materials significantly improved the separation performances of the membranes. Impressively, the as-obtained membrane acquired a high CO_2_ permeability of 119.08 Barrer and CO_2_/N_2_ selectivity of 76.26. Additionally, the presence of UiO-66@HNT conferred good long-term stability and excellent interfacial compatibility on the MMMs. The results demonstrated that the composite filler with fast transport pathways designed in this study was an effective strategy to enhance gas separation performance of MMMs, verifying its application potential in the gas purification industry.

## 1. Introduction

Due to the increasing consumption of fossil fuels by humans, the concentration of carbon dioxide in the atmosphere has led to a gradual global temperature increase. According to the Intergovernmental Panel on Climate Change (IPCC), CO_2_ levels will increase to 450 ppm in 2035, which will contribute to a 2 °C increase in global temperature [1]. Although global CO_2_ emissions dropped by 5.8% in 2020, the total amount of global CO_2_ emissions is still not optimal. As a new type of clean and efficient separation technology, membrane separation technology has developed rapidly because of its simplicity, low energy consumption, and easy combination with other separation methods. However, the current commercial polymer membranes exhibit unsatisfactory separation performances and a limitation called the trade-off effect, in which gas permeability and selectivity are difficult to achieve at the same time [2].

Researchers proposed to disperse organic/inorganic materials into polymers as fillers in order to obtain mixed matrix membranes (MMMs) [3,4]. Polymers would ensure preservation of the original excellent properties and low preparation cost, and the addition of inorganic fillers can effectively improve the permeability and selectivity of the membrane [5]. In MMMs, various fillers such as CNT, metal oxide, zeolites, and metal organic frameworks (MOFs) have been incorporated into polymers to fabricate membranes and utilize the extraordinary transport properties of the filler phase.

Metal organic frameworks (MOFs) are nanoporous materials composed of inorganic metal nodes coordinated with organic clusters [6]. MOFs have been used to great effect in a variety of applications due to their high porosity, adjustable pore structure, and rich chemical functions. Due to their uniform pore diameters, MOFs are also widely used for gas separations. Among various MOFs, UiO-66 is an MOF material with Zr as the metal center and benzene-1, 4-dicarboxylate (BDC) as bridged linkers [7,8]. It has a centric octahedral cage structure connected by eight tetrahedral cages (ca. 8 Å) and trigonal windows (ca. 6 Å) [9,10]. UiO-66 has a high specific surface area, higher porosity, and good thermal stability and chemical stability [11], making it a candidate for membrane gas separation. Chuah et al. [12] incorporated nonfunctionalized UiO-66 nanocrystals into a polyimide membrane and successfully improved CO_2_ permeability, with a slight decrease in CO_2_/N_2_ selectivity, owing to its large accessible surface area. The addition of other functional groups further improved the CO_2_/N_2_ selectivity of the polymeric membrane, with UiO-66-NH_2_, UiO-66-Br, and UiO-66-(OH)_2_ demonstrating improvements of 12%, 4%, and 17%, respectively. Sutrisna et al. [13] used UiO-66 to fabricate a nanocomposite hollow fiber membrane. The addition of nanofillers can effectively promote both CO_2_ permeance and selectivity. Due to good interfacial compatibility, the Pebax thin layer can host 50 wt.% UiO-66 without introducing extra defects and further increase the UiO-66 loading to 80 wt.% with only slightly reduced gas selectivity. Venna et al. [14] fabricated Matrimid-based MMMs dispersed in amine-based UiO-66 filler for gas separation. The results showed that, compared to pristine polymer, the permeability and selectivity were 200% and 23%, respectively, attributed to the molecular sieving ability and high porosity of UiO-66.

Halloysite nanotube (HNT) is an inorganic material which exists widely in nature. It is both environmentally friendly and cheaper than carbon nanotubes. Recently, due to the impact of carbon nanotubes on human health, HNT has been more widely adopted, as it is not a hazardous material that endangers human health. One application of HNTs is their use as filler in polymers to prepare MMMs [15]. HNT has a double-layer aluminosilicate structure and a hollow nanotube structure with open ends. Its chemical properties are similar to kaolin, but the crystal morphologies of the two structures are different [16]. The formula of HNT is [Al_2_Si_2_O_5_(OH)_4_·nH_2_O] [15]. The outer diameter is about 50–80 nm and the length varies from 300 nm to 1500 nm. The Al−OH structure is mainly distributed on the inner surface as an active group, making it easy to modify, and the outer surface is composed of siloxane (O-Si-O). In addition, HNT has a hollow tubular structure, which gives it a strong capacity for adsorption. At the same time, because of its hydrophilicity, HNT has good solubility in hydrophilic polymers [16]. Shi et al. [17] used HNT or SiO_2_ as a non-two-dimensional (2D) filler to be in pair with a 2D filler, MXene or graphene oxide (GO), to explore the underlying synergy between 2D nanosheets and a non-2D filler in MMMs for gas separation. By tuning the mass ratio of binary fillers, synergetic effect was found for each group of MMMs. The two 2D fillers found different preferential non-2D partners. GO works better with HNTs than SiO_2_, while MXene prefers SiO_2_ to HNTs. GO/HNTs MMMs achieved maximum enhancement of CO_2_ permeability (153%) and CO_2_/N_2_ selectivity (72%), as compared to the pristine Pebax membrane. Zhang et al. [18] demonstrated a method to template microphase separation through the controllable self-assembly of HNTs within the thin film via the solution casting method within a thin composite Pebax membrane. Crystallization of the polyamide component was induced at the HNT surface, guiding subsequent crystal growth around the tubular structure. The obtained composite membrane had an ultrahigh CO_2_/N_2_ selectivity (up to 290), being the highest selectivity recorded for Pebax-based membranes. Afshoun et al. [15] prepared Pebax/PEI (polyetherimide) composite membranes and investigated their CO_2_/CH_4_ gas separation performance. HNTs were added to Pebax layers at different loadings. The CO_2_ permeability increased from 3.25 to 4.2 GPU, but the CO_2_/CH_4_ selectivity decreased from 32 to 18, which was attributed to the finite pressure ratio of feed to permeate (i.e., 0.2) and the partial polymer swelling in the presence of CO_2_ gas, facilitating CH_4_ permeation.

In this study, an MOF-capped tubular structure was initially conceived following the solution-diffusion mechanism. A series of UiO-66-capped halloysite nanotubes (UiO-66@HNT) was designed and synthesized via a solvothermal method. UiO-66 was coated on HNT to create a pathway for gas transmission and accelerate CO_2_ into the lumen of HNT. Then, these nanotubes were embedded in the Pebax-1657 matrix to fabricate MMMs for CO_2_/N_2_ separation. Pebax-1657 is a commercial rubbery polymer used as continuous phase with high gas permeability and selectivity. It consists of a hard segment (PA) and a soft segment (PEO). The PEO has high CO_2_ permeability capability due to chain mobility and the PA segment shows strong mechanical strength [19,20]. The effects of different loadings and feed pressures on the CO_2_ permeability and CO_2_/N_2_ selectivity were also investigated. Finally, the long-term stability of the MMMs was studied in order to test their anti-aging behavior.

## 2. Materials and Methods

### 2.1. Materials

Halloysite nanotubes (HNTs) were supplied by Guangzhou Runwo Material Technology Co., Ltd. (Guangzhou, China), and were refined by sequentially milling, sieving, and drying under vacuum at 60 °C overnight. Dopamine hydrochloride (C_8_H_12_ClNO_2_, 98%) and zirconium (IV) chloride (ZrCl_4_ 98%) powder were supplied by Shanghai Aladdin Reagents Ltd. (Shanghai, China) Terephthalic acid (C_8_H_6_O_4_, 99%) was provided by Sigma-Aldrich (Shanghai, China). *N*,*N*-Dimethylformamide (DMF) was purchased by Sinopharm Chemical Reagent Co., Ltd. (Shenyang, China). Acetic acid (C_2_H_4_O_2_, 99%) was received from Xilong Chemical Co., Ltd. (Shantou, China). Pebax MH 1657 was purchased from Arkema (France). Research grade N_2_ and CO_2_ were supplied by Dalian Institute of Chemical Physics, Chinese Academy of Sciences (Dalian, China). Deionized water (DI water) was used in all experiments. All of the materials and solvents were used without further purification.

### 2.2. Synthesis of UiO-66@HNT Composite Materials

The HNTs were first treated with dopamine hydrochloride to obtain the polydopamine layer (PHNTs), which has high affinity for zirconium ions [21,22]. HNTs (1.0 g) were dispersed into the water (100 mL), then dopamine hydrochloride (0.1 g) was dissolved into the mixture. The mixture was stirred for 30 min. Subsequently, 8.44 mL of KOH solution (1 mol/L) was rapidly injected into the mixture. The samples were kept under stirring without perturbation at room temperature for 30 min, then centrifuged to collect PHNTs for subsequent use.

**Figure 1:**
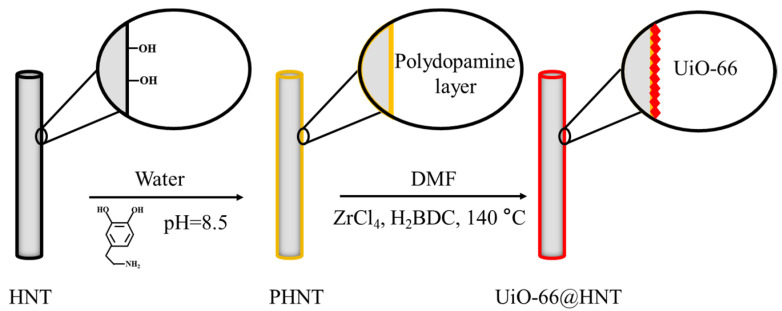
The preparation procedure of UiO-66@HNT.

The preparation procedure of UiO-66@HNT nanotubes is illustrated in Figure 1. The as-prepared PHNTs (0.5 g) were dispersed in a DMF solution of ZrCl_4_ (1.16 g, 5 mmol) and then redispersed in a DMF solution of terephthalic acid (0.83 g, 5 mmol). The mixture was stirred for 1 h, and then, 3 mL of acetic acid was dissolved in the mixture and heated at 140 °C for 24 h. The product was obtained by centrifugation at 5000× *g* rpm for 5 min, washed with DMF and water three times, and dried overnight in the oven at 60 °C.

### 2.3. Fabrication of MMMs

MMMs and pristine Pebax membranes were fabricated by the solution-casting method. Pebax-1657 9.325 g pellets were dissolved in a mixture of ethanol/deionized water (210 g ethanol, 90 g deionized water) under reflux for 4 h at 80 °C to obtain a 3 wt.% homogeneous solution. Simultaneously, a specified amount of UiO-66@HNT was dispersed into the Pebax solution and kept stirring for 30 min. After being stirred, the mixture was placed under ultrasonic treatment for another 2 h to remove bubbles. Then, the UiO-66@HNT/Pebax solutions were poured into plastic petri dishes and dried at 40 °C for 24 h in the oven to remove residual solvent.

The UiO-66@HNT loading is defined as

$$\mathrm{UiO}-66@\mathrm{HNT}\;\mathrm{loading}\;(\mathrm{wt}.\%)=\frac{m_{UiO-66@HNT}}{m_{UiO-66@HNT}+m_{Pebax}}\times 100\%$$

where $m_{UiO-66@HNT}$ and $m_{Pebax}$ are the mass of UiO-66@HNT and Pebax. 

**Figure 2:**
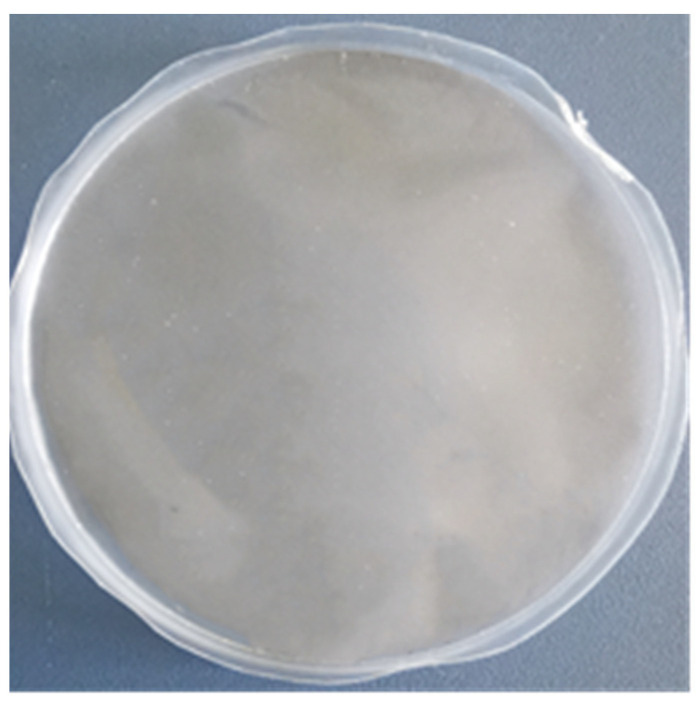
Image of UiO-66@HNT/Pebax mixed matrix membrane.

In this work, pure Pebax membrane and UiO-66@HNT/Pebax MMMs with different loadings of 5, 10, 15, and 20 wt.% were prepared. The membranes are shown in Figure 2. The thickness of each was about 75–90 μm.

### 2.4. Characterization

To characterize these materials, a series of tests was performed. Morphologies of UiO-66@HNT were characterized using transmission electron microscopy (TEM) on a Tecnai G2 F30 S-Twin from Thermo Scientific (America). The size and morphology of UiO-66@HNT and the dispersion of the filler in the membranes were characterized with Nova Nano SEM 450 scanning electron microscopy (SEM) at 20 kV (America). The cross-sections were prepared under liquid N_2_. X-ray diffraction (XRD) data of all materials and membranes were obtained from 5 °C to 90 °C at a scanning rate of 10° in Rigaku SmartLab 9kw (Japan). Fourier transform infrared (FTIR) spectra were obtained using a MAGNA-560 spectrometer manufactured by the Bruker Company (German). The spectra were obtained in the range of wavenumbers from 4000 to 400 cm^−1^ with a scan per sample. Nitrogen adsorption–desorption isotherms were used to characterize the surface area of UiO-66@HNT and measured at −196 °C using a porosity analyzer (Autosorb iQ) (America). Thermo gravimetric analyses (TGA) were performed using a Mettler Toledo TGA/ SDT 851^e^ system. (America) Samples were heated in N_2_ flow up to 800 °C at a heating rate of 10 °C/min. DSC curves were obtained from −60 °C to 250 °C at a heating rate of 10 °C/min from American TA Company 2500 (America).

### 2.5. Permeability Experiment

Pure gas (CO_2_, N_2_) permeation experiments were conducted using a constant volume gas permeation device [23,24]. After the test membrane was installed into the membrane module, it was degassed for at least 8 h and then subjected to leakage tests of the membrane. Then, the permeability coefficient of CO_2_ and N_2_ was tested using the equal volume–variable pressure method, for which the specific formula follows:

$$P=\frac{VL}{ART\left(p_{1}-p_{2}\right)}\left(\frac{dp_{2}}{dt}\right)$$

where *P* is the permeability of N_2_ and CO_2_ (Barrer, 1 Barrer = 10^−10^ cm^3^(STP) cm cm^−2^ s^−1^ cmHg^−1^), *L* is the thickness of the membrane (cm), *V* is the volume of the permeate side of the membrane (cm^3^), $p_{1}$ is the pressure of the feed side (cmHg) and $p_{2}$ is the pressure of the permeate side (cmHg), *A* is the effective membrane area (cm^2^), *T* is the absolute temperature (K), $\frac{dp_{2}}{dt}$ is pressure rise rate (cmHg s^−1^), and *R* is the ideal gas constant (0.278 cmHg cm^3^ cm^−3^(STP) K^−1^).

The CO_2_/N_2_ selectivity ($\alpha_{CO_{2}/N_{2}}$) was calculated by:

$$\alpha_{CO_{2}/N_{2}}=\frac{P_{CO_{2}}}{P_{N_{2}}}$$

A lag time method [25,26] was used to calculate the gas diffusion coefficient (*D*) and solubility coefficient (*S*) of the membrane through the dissolution–diffusion mechanism:

$$D=\frac{L^{2}}{6\theta }$$

$$S=\frac{P}{D}$$

where *θ* is the gas diffusion lag time, *S*, and *L* is the membrane thickness, cm.

### 2.6. Maxwell Model

The gas permeabilities of UiO-66@HNT/Pebax were backcalculated via the Maxwell model, which is one of the most widely used models for composite materials to predict permeabilities:

$$P_{MMM}=P_{P}\left[\frac{P_{S}+2P_{P}-2\Phi_{S}\left(P_{P}-P_{S}\right)}{P_{S}+2P_{P}+\Phi_{S}\left(P_{P}-P_{S}\right)}\right]$$

where $P_{MMM}$ is the MMM’s permeability,$\;P_{P}$ is the pristine Pebax permeability, $P_{S}\;$ is the dispersed phase permeability (UiO-66@HNT), and $\Phi_{S}$ is the volume fraction of molecular sieves in the polymer phase [27,28]. In this work, UiO-66@HNT molecular sieves’ permeability was estimated to be 794 Barrer via the measurement of dissolution–diffusion coefficients.

The formula of volume fraction of UiO-66@HNT in the MMMs is:

$$\Phi_{S}=\frac{m_{s}/\rho_{s}}{m_{s}/\rho_{s}+m_{c}/\rho_{c}}$$

where $m_{s}$ and $\rho_{s}\;$ are the mass and density of the filler phase (UiO-66@HNT) (0.43 g cm^−3^), and $m_{c}$ and $\rho_{c}$ are the mass and density of the polymer phase (Pebax) (0.99 g cm^−3^). In most cases, the void volume can be neglected [29]. Consequently, the apparent volume fraction was nearly equal to the UiO-66@HNT volume fraction in the MMMs.

## 3. Results

### 3.1. FTIR of UiO-66@HNT and the UiO-66@HNT/Pebax Membrane

**Figure 3:**
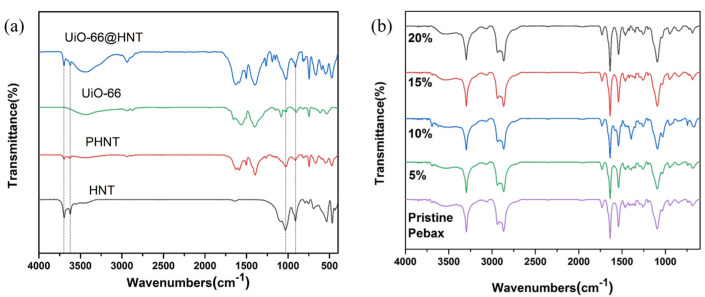
FTIR spectrum of: (**a**) HNT, PHNT, UiO-66, and UiO-66@HNT; (**b**) Pristine Pebax and UiO-66@HNT/Pebax MMMs with different loadings.

FTIR analyses of HNT, PHNT, UiO-66, and UiO-66@HNT were performed and are shown in Figure 3a. In the spectrum of HNT, two bands appeared at about 3610 cm^−1^ and 3640 cm^−1^, associated with ‒OH, and two characteristic bands at 1031 cm^−1^ and 908 cm^−1^ appeared in the spectrum of pristine HNTs, which were ascribed to the bending of Si–O–Si stretching and –Al–O deformation. These four bands also appeared in the spectrum of PHNT and UiO-66@HNT, and they did not appear in the spectrum of UiO-66. It was proven that UiO-66@HNT and PHNT had the same structure as HNT. A band at about 2850 cm^−1^ appeared in the spectrum of UiO-66. This peak also appeared in the spectrum of UiO-66@HNT but did not appear in the spectrum of HNT and PHNT, showing that the UiO-66@HNT composite material contained the same structure as UiO-66.

FTIR analyses of pristine Pebax and UiO-66@HNT/Pebax MMMs with different filler loadings are shown in Figure 3b. As shown, there was no appearance of new bands because the dispersed phase (UiO-66@HNT) and continuous phase (Pebax) were a physical blend. Thus, the mixing of UiO-66@HNT composite material did not destroy the inner structure of the MMMs, and the composite material was perfectly integrated into the matrix of the MMMs.

### 3.2. XRD of UiO-66@HNT and the UiO-66@HNT/Pebax Membrane

**Figure 4:**
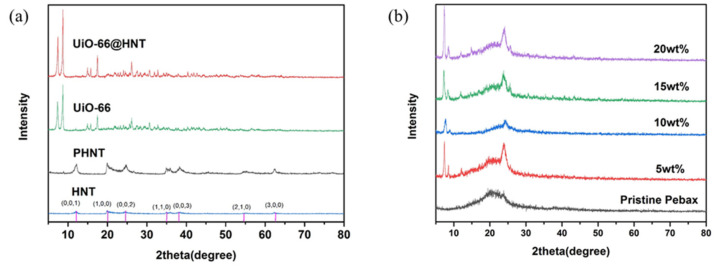
XRD patterns of: (**a**) HNT, PHNT, UiO-66, and UiO-66@HNT; (**b**) Pristine Pebax and MMMs with different loadings.

The crystal structure of the fillers is presented in Figure 4. As shown in Figure 4a, the XRD pattern of HNT had a diffraction peak in 2θ = 12.11°, 20.07°, and 24.57°. These diffraction peaks were also shown in the XRD patterns of PHNT and UiO-66@HNT at the same time. The XRD pattern of UiO-66 had sharp diffraction peaks at 2θ = 7.36° and 8.48°. Two characteristic peaks of UiO-66 also appeared in the XRD pattern of UiO-66@HNT, which corroborated the combination of HNTs and UiO-66. Additionally, the incorporation of UiO-66 did not destroy the crystal structure.

The XRD patterns of MMMs can reflect the crystal structures of the dispersed phase and the polymer phase. As shown in Figure 4b, the broader peak at 20.0° illustrated the soft phase of PEO, and the distinct peak at 24.0° indicated the crystalline PA phases. Compared with the pristine Pebax membrane, the XRD pattern of the MMMs also showed characteristic peaks based on UiO-66@HNT, indicating that the filler was successfully dispersed in the Pebax without changing the crystal structure of the filler.

### 3.3. The Characterization of UiO-66@HNT and the UiO-66@HNT/Pebax Membrane

**Figure 5:**
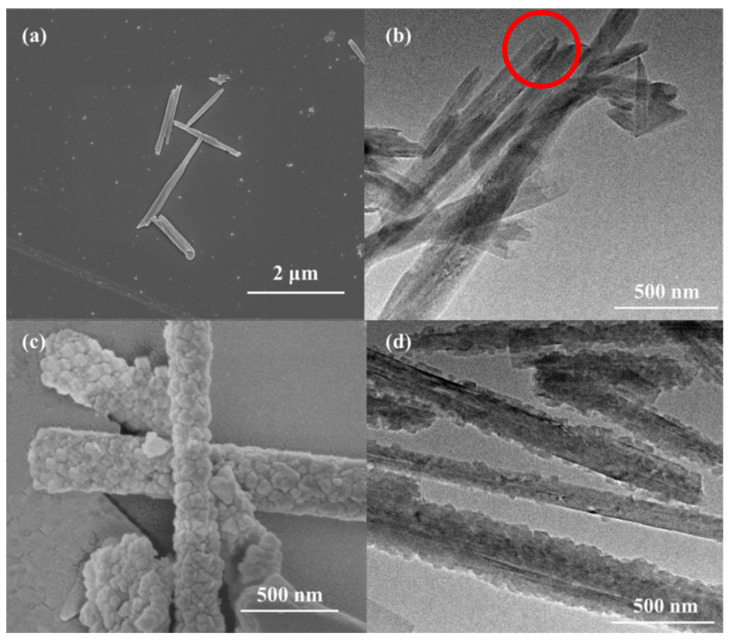
SEM image and TEM image of: (**a**,**b**) HNT and; (**c**,**d**) UiO-66@HNT.

The morphologies of the as-prepared materials and membranes were characterized using SEM and TEM. As shown in Figure 5, the pristine HNT was relatively smooth; the inner diameter of HNT was about 22 nm. It can be seen from the red circle in Figure 5b that the top of the pure HNT was open and had a hollow tubular structure. Compared with the pristine HNT in Figure 5c, the surface was rough and covered with a crystal structure. The rough surface is shown in Figure 5d and Figure S1. The UiO-66 layer not only appeared at the external surface, but also at the open-ending pores of HNTs. A possible explanation was that both the lumen and external surfaces of HNTs could be deposited by the polydopamine layer, which induced the heterogeneous nucleation of UiO-66 [30], indicating that the UiO-66 was coated on HNT and the UiO-66@HNT composite material was successfully prepared.

**Figure 6:**
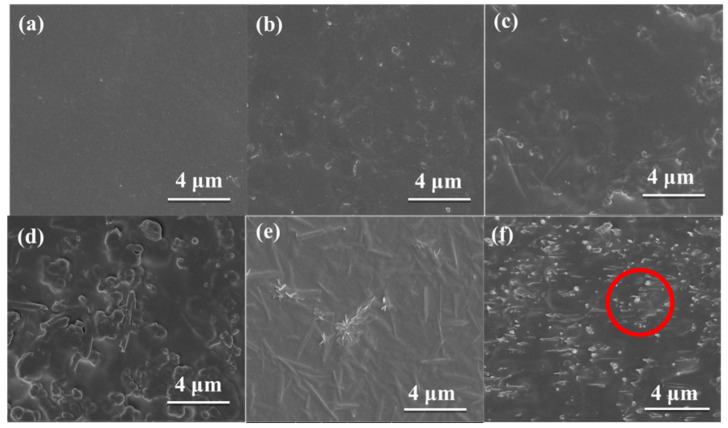
SEM images of MMMs with different loadings: (**a**) 0 wt.%; (**b**) 5 wt.%; (**c**) 10 wt.%; (**d**) 15 wt.%; (**e**) 20 wt.%, and; (**f**) cross-section image of 20 wt.% MMM.

SEM images of pristine Pebax and UiO-66@HNT/Pebax membranes are shown in Figure 6. The pristine Pebax membrane was relatively smooth and dense without any agglomerations. However, the MMMs began to show white spots compared with the pristine Pebax under a loading of 5 wt.%, which was the end of the tubular structure of UiO-66@HNT. As shown in Figure 6b–e, the UiO-66@HNT composite materials were well dispersed in the matrix with no defects at the interface and without agglomeration of the dispersed phase. This was because the UiO-66 on the surface of the UiO-66@HNT composite material had good affinity with the matrix, which prevented agglomeration from occurring. EDS mapping was used to scan the Zr element in UiO-66@HNT. It can be seen from Figure S2 that the composite materials were well dispersed in the MMM. When the loading of the UiO-66@HNT composite material increased, the surface grew rough and the number of white spots increased. The cross-section image of UiO-66@HNT/Pebax membranes is also shown in Figure 6f and Figure S3. With the mixing of UiO-66@HNT, the cross-section of the MMM showed a certain number of stripes but remained dense. In addition, the tubular structure can be seen in the red circle in Figure 6f, which is the same as that of UiO-66@HNT in Figure 5b, indicating that the UiO-66@HNT composite material was successfully dispersed into the MMM. This result confirmed that the presence of UiO-66 on the surface of HNT improved the interfacial compatibility between the filler and the polymer matrix.

### 3.4. TGA of the Composite Material

**Figure 7:**
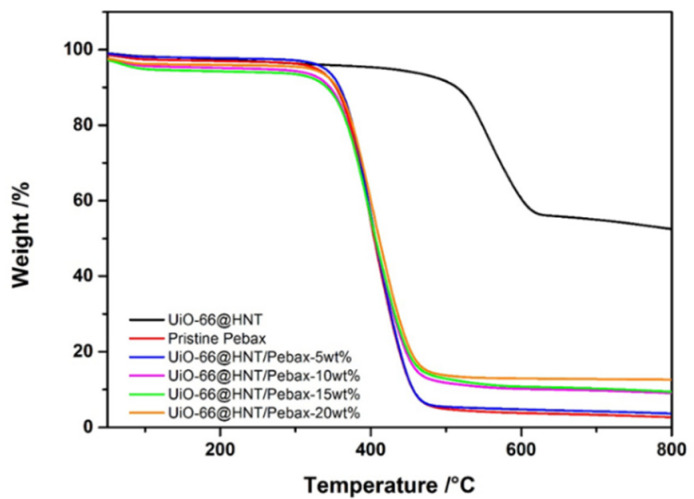
TGA curves of UiO-66@HNT and UiO-66@HNT/Pebax membranes.

The TGA curves of the UiO-66@HNT composite material and the membranes are shown in Figure 7. In the range of 30–500 °C, the composite material experienced slight mass loss, mainly the residual or adsorbed moisture in the sample. Then, the curve experienced significant mass loss starting from 500 °C, which is attributable to the collapse and decomposition of the UiO-66 framework structure capped on the surface of HNT. Additionally, the organic ligands gradually pyrolyzed and evaporated because HNT’s chemical properties were similar to kaolin; it did not decompose and only the non-decomposed ZrO_2_ and HNT remained.

Next, the TGA curves of MMMs were observed to determine whether the introduction of composite materials would affect thermal stability. As can be seen in Figure 7, the thermal stability of the MMMs did not change with the addition of composite materials. The main thermal decomposition occurred between 350 and 450 °C, and the mass loss came from the decomposition of the Pebax polymer backbone. Due to the low loadings during the thermal decomposition of the material, the downward trend of the curve was not obvious in the range of 600–700 °C. Besides, the glass transition temperature (T_g_) and melting point (T_m_) of the crystalline region of the MMMs were measured by DSC. The results are shown in Figure S4 and Table S1. It can be seen that the introduction of the composite materials in the MMMs did not change the melting points of the PEO and PA segments.

### 3.5. BET Characterization of the Material

**Figure 8:**
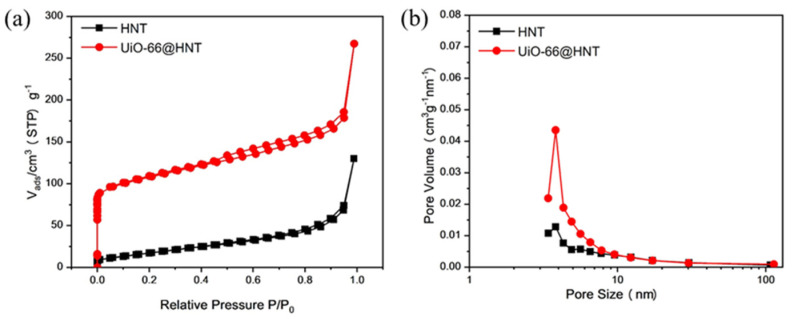
BET data of HNT and UiO-66@HNT: (**a**) Nitrogen adsorption–desorption curve; (**b**) Pore size distribution curve.

As shown in Figure 8, HNT exhibited type II isotherms with H3 hysteresis loops and UiO-66@HNT exhibited a combination of hybrid type I/IV isotherms with H3 hysteresis loops, which indicated that the samples contained mesoporous structures. As shown in Table 1, the BET surface area of UiO-66@HNT was about 395.06 m^2^ g^−1^, which was much larger than that of HNTs (62.56 m^2^ g^−1^). The increasing UiO-66 content in the HNT contributed to this tendency. The pore size distribution curve of the HNT and composite material UiO-66@HNT exhibited a mesoporous structure. The mesopores were attributed to the mesoporous lumen of HNTs in the composite materials. Besides, the MOF layer only slightly reduced the HNTs’ Brunauer–Joyner–Halenda (BJH) pore size and increased the Brunauer–Emmett–Teller (BET) surface area, confirming a very thin UiO-66 layer.

### 3.6. Gas Permeation Measurements

#### 3.6.1. Effect of Filler Type and Content on Gas Separation Performance

**Figure 9:**
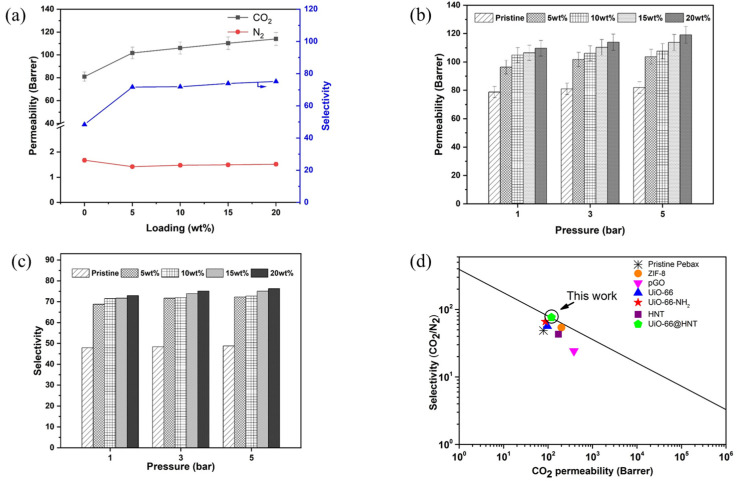
Gas permeation tests of UiO-66@HNT/Pebax MMMs: (**a**) CO_2_, N_2_ permeability and CO_2_/N_2_ selectivity of the membranes with different UiO-66@HNT loadings (0, 5 wt.%, 10 wt.%, 15 wt.%, and 20 wt.%); (**b**) CO_2_ permeability in different pressure; (**c**) CO_2_/N_2_ selectivity in different pressure; (**d**) the relationship between the CO_2_ permeability and the CO_2_/N_2_ selectivity of the MMMs prepared in this work and in the literature.

CO_2_ and N_2_ permeation tests were performed to research the effects of UiO-66@HNT filler loading on the gas separation performance of MMMs. Figure 9a shows the gas permeability and selectivity of the MMMs obtained by the average values of at least three permeation tests. As shown, the error of gas permeability was small, confirming the repeatability of the results. The results showed that the CO_2_ permeability and CO_2_/N_2_ selectivity of the pristine Pebax were 80.97 Barrer and 48.37, respectively. It could also be seen that the CO_2_ permeability of the UiO-66@HNT/Pebax MMM increased from 80.97 to 113.94 Barrer when the filler loading increased from 0 wt.% to 20 wt.%. Additionally, the N_2_ permeability increased from 1.41 to 1.67 Barrer, but the rate of increase was slower than in CO_2_. This enhancement of CO_2_ and N_2_ was attributed to the microstructure of the UiO-66@HNT. The combination of HNT and UiO-66 increased the free volume of the Pebax. The UiO-66@HNT/Pebax membrane had a higher CO_2_/N_2_ selectivity than the pristine Pebax membrane because of the filler creating a pathway for CO_2_ transmission. Therefore, the gas separation properties of the UiO-66@HNT/Pebax membrane were better than the pristine Pebax membrane.

Detailed analysis on gas diffusivity and solubility coefficients of UiO-66@HNT/Pebax membranes are summarized in Table 2. When the filler loading was increased from 0 to 20 wt.%, the diffusivity coefficients of CO_2_ increased substantially, indicating the increase in CO_2_ selectivity was mainly due to the improvement in diffusivity selectivity for CO_2_. The results indicate that the addition of fillers increases the free volume in the membrane, and the free volume increases as the filler loading increases. By adding fillers into the polymer matrix, disturbed polymer chain packing and increased HNT/polymer interfacial volume can be created, thereby increasing gas diffusivity via introducing more alternative routes for passing through gas molecules.

Furthermore, the Maxwell model was used to predict the gas permeation properties of the MMMs in this work. Figure S5 shows the CO_2_ and the N_2_ permeability of the MMMs predicted by the Maxwell model and both were in agreement with the experimental permeation results (within the available error range). Therefore, we could use this model to predict the gas performance of the mixed matrix membranes with higher loadings.

#### 3.6.2. Effect of Feed Pressure on Gas Separation Performance

Figure 9b,c show the effects of feed pressure on gas permeability and selectivity of the pristine Pebax and UiO-66@HNT/Pebax MMMs. The other results are shown in Table S2. Generally, the separation principle of the rubbery polymer membrane is the dissolution and diffusion mechanism. The increase in pressure will increase the solubility and diffusion rate of the gas in the membrane, respectively. Therefore, the permeability of the two gases was improved to a certain extent. As the feed pressure increased from 1 to 5 bars, the CO_2_ permeability of the membrane samples showed an increased trend. With the incorporation of UiO-66@HNT materials, the permeability and selectivity still increased with the increased pressure. Its incorporation exhibited a significant effect on the gas separation performance.

#### 3.6.3. Comparison with Robeson’s Upper Bound

The CO_2_ permeability and selectivity of synthetic MMM were compared with the Robeson’s upper bound established in 2008 [2]. When the filler loading of UiO-66@HNT was 20 wt.%, the permeability and the selectivity of the MMM were close to the Robeson upper bound (2008) as shown in Figure 9d. The permeability and selectivity of the UiO-66@HNT/Pebax MMM were much better than for the pristine Pebax membrane. The CO_2_ separation performance of Pebax-1657-based MMMs in reported literature compared with this work is listed in Table 3. Based on the above results, the improvement of separation performance with the UiO-66@HNT/Pebax membrane confirmed that the addition of the UiO-66@HNT composite enhanced the interface morphology and gas separation performances.

#### 3.6.4. The Mechanism of UiO-66@HNT

**Figure 10:**
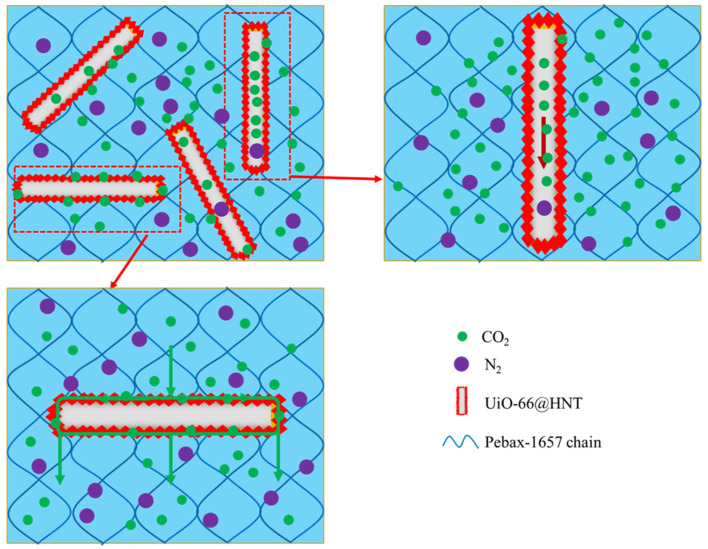
Schematic diagram of the gas permeation mechanism of UiO-66@HNT/Pebax membranes.

The mechanism of UiO-66@HNT is shown in Figure 10. In this work, UiO-66@HNT was a new type of material that combined MOF material UiO-66 with natural halloysite nanotubes. The HNT had a hollow nanotube structure with open ends, and the resistance to gas in the closed tube was less than that outside the tube, making HNT an excellent molecular transport channel. When the material was arranged vertically, it promoted the diffusion of molecules and improved the permeability of the MMM. In addition, UiO-66 had a high affinity for CO_2_. UiO-66 was coated on HNT so that CO_2_ could accelerate into the lumen of HNT under the attraction of UiO-66. Then, the UiO-66 on the outer surface of the HNT formed a continuous layer, which provided a pathway for gas transmission when the composite material was arranged horizontally. In this way, the permeability of the membrane to CO_2_ was accelerated so that the selectivity of CO_2_/N_2_ was increased.

#### 3.6.5. Long-Term Stability of MMMs with UiO-66@HNT

**Figure 11:**
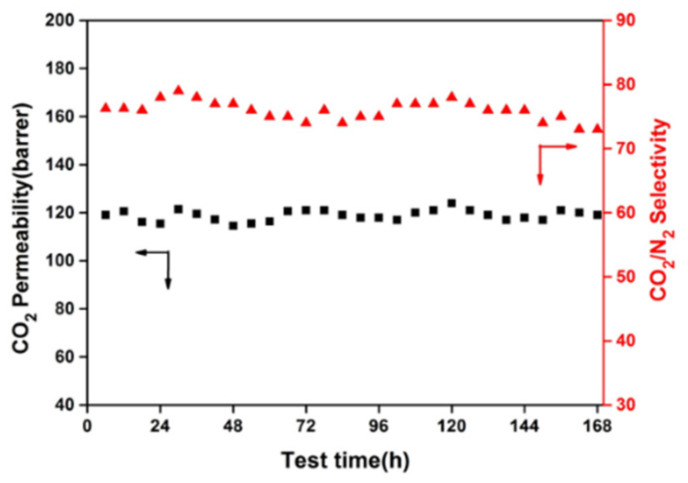
The stability test of the UiO-66@HNT/Pebax (20 wt.%) at 25 °C and 5 bars (feed pressure).

The anti-aging behavior of MMMs is very crucial in practical applications [34,35]. Thus, the long-term stability of the membranes with optimum performance was tested, as shown in Figure 11. The membrane still had stable CO_2_ permeability and CO_2_/N_2_ selectivity under the feed pressure of 5 bars for 168 h. The CO_2_ permeability and CO_2_/N_2_ selectivity were basically unchanged, indicating excellent anti-aging behavior and structural stability (up to 120 h) [36,37,38].

## 4. Conclusions

In summary, UiO-66@HNT, a novel composite material, was designed and synthesized via a solvothermal method, after which various loadings of UiO-66@HNT/Pebax MMMs using the solution casting and solvent evaporation methods were prepared and applied in gas separation. FTIR, XRD, SEM, TGA, and BET measurements were conducted to characterize the structure and morphology of the prepared membranes. UiO-66@HNT exhibited outstanding compatibility with the Pebax matrix. For the gas separation process, UiO-66@HNT/Pebax membranes showed an obvious enhancement of gas separation performance compared with the pristine Pebax membrane. When 20 wt.% UiO-66@HNT was prepared, the fillers exhibited the highest permeability and selectivity of 119.08 Barrer and 76.26, respectively. In addition, the long-term stability of the MMMs exhibited stable gas permeability and outstanding anti-aging properties. The results demonstrated that the designed composite filler with fast transport pathways was an effective strategy to enhance the gas separation performance of MMMs, verifying their application potential in the gas purification industry.

## Figures and Tables

**Table 1:** BET data of HNT and UiO-66@HNT.

Sample	HNT	UiO-66@HNT
BET surface/m^2^∙g^−1^	62.56	395.06
Pore volume/cm^3^∙g^−1^	0.201	0.413
Average pore size/nm	3.83	3.82

**Table 2:** Gas diffusivity coefficients and solubility coefficients of UiO-66@HNT/Pebax membranes.

UiO-66@HNT Loading (wt.%)	D(CO_2_) ^a^	S(CO_2_) ^b^	D(N_2_) ^a^	S(N_2_) ^b^
0	10.43 ± 0.03	7.76 ± 0.03	7.61 ± 0.04	0.20 ± 0.01
5	12.51 ± 0.02	8.14 ± 0.02	6.69 ± 0.03	0.21 ± 0.02
10	12.94 ± 0.04	8.22 ± 0.05	6.38 ± 0.05	0.24 ± 0.01
15	13.32 ± 0.06	8.29 ± 0.02	5.98 ± 0.03	0.25 ± 0.01
20	13.84 ± 0.03	8.26 ± 0.04	5.77 ± 0.06	0.26 ± 0.02

^a^ Diffusivity coefficient [cm^2^/s] × 10^6^, ^b^ Solubility coefficient [cm^3^(STP)/cm^3^ cmHg] × 10^4^.

**Table 3:** Comparison of the separation performance of other MMMs based on Pebax substrates in the literature with our current work under dry conditions.

Materials	Conditions	CO_2_ Permeability (Barrer)	CO_2_/N_2_ Selectivity	Refs.
Pebax-1657	5 bar, 25 °C	78.6	48.7	This work
HNT/Pebax-1657	2 bar, 30 °C	171	43	[17]
ZIF-8/Pebax-1657	-	199.57	53.88	[31]
pGO/Pebax-2533	1 bar, 35 °C	380.44	24.19	[32]
UiO-66/PEBA	3 bar, 20 °C	96.3	56.6	[33]
UiO-66-NH_2_/PEBA	3 bar, 20 °C	87.0	66.1	[33]
UiO-66@HNT/Pebax-1657	5 bar, 25 °C	119.08	76.26	This work

## Supplementary Materials

The following are available online at https://www.mdpi.com/article/10.3390/membranes11090693/s1 Figure S1. TEM images of UiO-66@HNT, Figure S2. SEM image and EDS mapping (Zr) of UiO-66@HNT/Pebax MMM, Figure S3. The cross-section SEM images of MMMs with different loadings (a,b) 5 wt.% (c,d) 10 wt.% (e,f) 15 wt.%, Figure S4. DSC curves of MMMs with different loadings, Table S1. Thermal properties of Pebax and corresponding mixed matrix membranes, Figure S5. Experimental and Maxwell model predicted permeation properties of UiO-66@HNT/Pebax mixed matrix membranes, Table S2. CO_2_ permeability and CO_2_/N_2_ selectivity of MMMs with different loadings and different pressures
