# Supplementary material for: A Novel Composite Material UiO-66@HNT/Pebax Mixed Matrix Membranes for Enhanced CO2/N2 Separation
Source: Membranes (Basel). 2021 Sep 7;11(9):693. doi: 10.3390/membranes11090693 (PMC8467370; doi:10.3390/membranes11090693)
Supplement: Supplementary file 1 [file membranes-11-00693-s001.zip › membranes-1316587-supplementary.pdf]

Supporting information

A novel composite material UiO-66@HNT/Pebax mixed matrix membranes for enhanced CO_2/N_2 separation

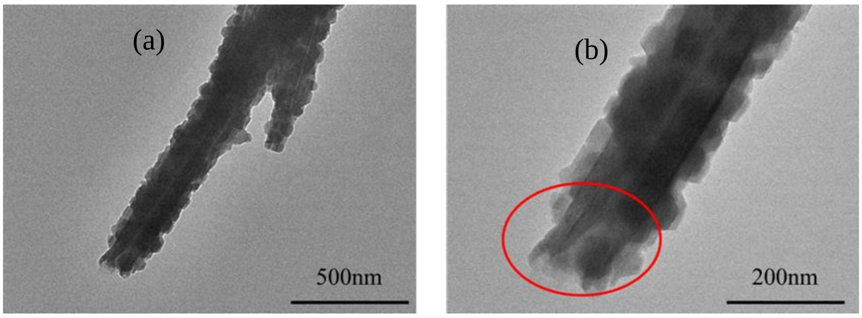

Figure S1. TEM images of UiO-66@HNT

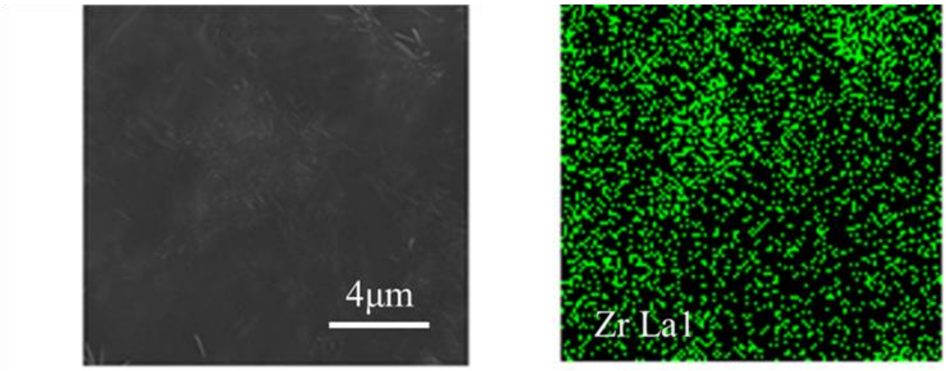

Figure S2. SEM image and EDS mapping (Zr) of UiO-66@HNT/Pebax MMM.

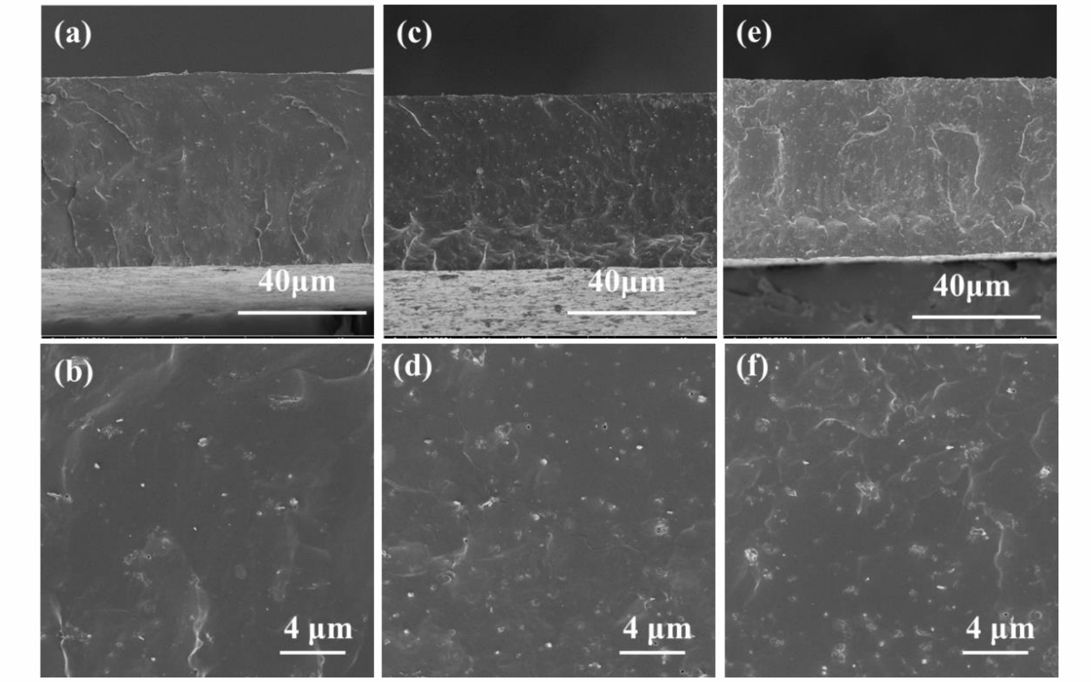

Figure S3. The cross-section SEM images of MMMs with different loadings (a, b) 5wt%, (c, d) 10wt% (e, f) 15wt%

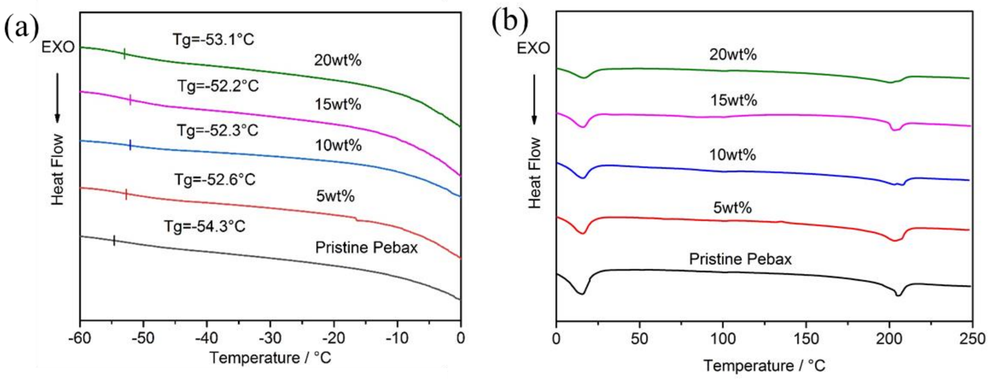

Figure S4. DSC curves of MMMs with different loadings

Table S1. Thermal properties of Pebax and corresponding mixed matrix membranes.

Membranes	T_g (°C)	PEO	PA
		T_g (°C)	T_m (°C)
**Pebax**	-54.3	15.41	204.87
**5wt%**	-52.6	15.04	203.02
**10wt%**	-52.3	15.49	204.27
**15wt%**	-52.2	15.83	202.70
**20wt%**	-53.1	15.77	203.27

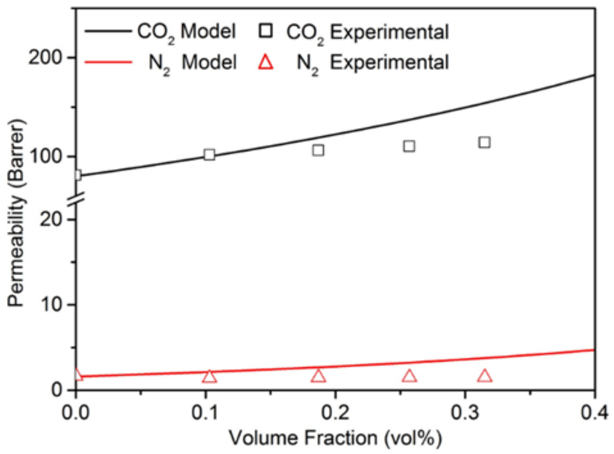

Figure S5. Experimental and Maxwell model predicted permeation properties of UiO-66@HNT/Pebax mixed matrix membranes.

Table S2. CO_2 permeability and CO_2/N_2 selectivity of MMMs with different loadings and different pressures

Loadings (wt%)		Pressure (bar)		
		1	3	5
0	P_{CO2}	78.73	80.97	81.97
	$S_{CO2/N2}$	47.98	48.37	48.79
5	P_{CO2}	96.33	101.71	103.70
	$S_{CO2/N2}$	68.79	71.69	72.26
10	P_{CO2}	104.84	106.06	107.55
	$S_{CO2/N2}$	71.52	71.87	72.69
15	P_{CO2}	106.44	110.28	113.74
	$S_{CO2/N2}$	71.69	73.91	75.05
20	P_{CO2}	109.64	113.94	119.07
	$S_{CO2/N2}$	72.93	75.11	76.25
